# GPS-PBS: A Deep Learning Framework to Predict Phosphorylation Sites that Specifically Interact with Phosphoprotein-Binding Domains

**DOI:** 10.3390/cells9051266

**Published:** 2020-05-20

**Authors:** Yaping Guo, Wanshan Ning, Peiran Jiang, Shaofeng Lin, Chenwei Wang, Xiaodan Tan, Lan Yao, Di Peng, Yu Xue

**Affiliations:** Key Laboratory of Molecular Biophysics of Ministry of Education, Hubei Bioinformatics and Molecular Imaging Key Laboratory, Center for Artificial Intelligence Biology, College of Life Science and Technology, Huazhong University of Science and Technology, Wuhan 430074, Hubei, China; guoyaping@hust.edu.cn (Y.G.); ningwanshan@hust.edu.cn (W.N.); peiran@hust.edu.cn (P.J.); linshaofeng@hust.edu.cn (S.L.); wangchenwei@hust.edu.cn (C.W.); tanxiaodan@hust.edu.cn (X.T.); yaolan@hust.edu.cn (L.Y.); pengdi@hust.edu.cn (D.P.)

**Keywords:** protein phosphorylation, phosphoprotein-binding domain, phosphorylation site, PPBD-specific binding p-site, deep learning, protein kinase

## Abstract

Protein phosphorylation is essential for regulating cellular activities by modifying substrates at specific residues, which frequently interact with proteins containing phosphoprotein-binding domains (PPBDs) to propagate the phosphorylation signaling into downstream pathways. Although massive phosphorylation sites (p-sites) have been reported, most of their interacting PPBDs are unknown. Here, we collected 4458 known PPBD-specific binding p-sites (PBSs), considerably improved our previously developed group-based prediction system (GPS) algorithm, and implemented a deep learning plus transfer learning strategy for model training. Then, we developed a new online service named GPS-PBS, which can hierarchically predict PBSs of 122 single PPBD clusters belonging to two groups and 16 families. By comparison, GPS-PBS achieved a highly competitive accuracy against other existing tools. Using GPS-PBS, we predicted 371,018 mammalian p-sites that potentially interact with at least one PPBD, and revealed that various PPBD-containing proteins (PPCPs) and protein kinases (PKs) can simultaneously regulate the same p-sites to orchestrate important pathways, such as the PI3K-Akt signaling pathway. Taken together, we anticipate GPS-PBS can be a great help for further dissecting phosphorylation signaling networks.

## 1. Introduction

In eukaryotes, protein phosphorylation is by far the most important and widespread post-translational modification that mainly occurs on specific serine (S), threonine (T) or tyrosine (Y) residues in protein substrates, and orchestrates a variety of biological processes including signaling transduction, cell cycle/proliferation, autophagy and metabolism [1,2,3,4]. Importantly, numerous proteins containing phosphoprotein-binding domains (PPBDs) can recognize and bind phosphoserine (pS), phosphothreonine (pT) or phosphotyrosine (pY) residues in specific substrates as “readers”, which dictate the phosphorylation signaling events delivered from “writers”, namely, protein kinases (PKs), and accurately propagate signals into downstream pathways [5,6,7]. Dysregulation of normal interactions between PPBDs and p-sites is frequently associated with human diseases such as cancer [8,9] and neurodegenerative disorders [10]. Thus, the identification of PPBD-specific binding p-sites (PBSs) is fundamental for revealing dynamic phosphorylation signaling networks.

In 1987, the Tony Pawson group discovered the first PPBD named Src homology 2 (SH2) domain that could bind to a broad range of pY-containing proteins [11,12]. Subsequently, a variety of experimental methods, such as including phage display, one-peptide/one-pin type techniques, alanine-scanning mutagenesis of the protein substrates, and oriented peptide library screening (OPLS), were established to identify phosphorylation-mediated interactions (PMIs) and/or pinpoint the exact PBSs [13]. For example, Keilhack et al. used the alanine-scanning mutagenesis approach to identify the pY1173 of the epidermal growth factor receptor (EGFR) as the major PBS of SHP-1/PTPN6, a SH2-containing protein-tyrosine phosphatase [14]. In 2003, Elia et al. adopted the OPLS approach to define a core consensus motif S-(pT/pS)-(P/X) recognized by the polo-box domain (PBD) of the mitotic kinase polo-like kinase 1 (PLK1) [15]. They found this motif to be present in a number of PLK1 substrates, and validated pT130 of Cdc25C as a novel PBS of PLK1-PBD [15]. Besides the conventional methods, co-immunoprecipitation coupled to mass spectrometry has turned to be a high-throughput approach for large-scale identification of PMIs and PBSs. Using this approach, Lowery et al. identified 622 potential PLK1-interacting proteins, and further detected 53 potential PBSs for PLK1-PBD [16]. 

Due to data accumulation, several databases were developed to maintain known PMIs and PBSs [17,18,19,20]. For example, Gong et al. developed a highly useful database named PepCyber:P∼PEP, which curated 7044 known PMIs between 337 PPBD-containing proteins (PPCPs) belonging to 10 families and 1123 interacting proteins [17]. In addition, Tinti et al. collected ~300 14-3-3-binding p-sites, and constructed a data resource called ANIA to annotate the 14-3-3 interactome [19]. Two databases HPRD and Phospho.ELM for more general purposes also contained 105 and 220 PMIs, respectively [18,21]. 

In contrast to labor-intensive and time-consuming experiments, computational prediction of PBSs from protein sequences is an alternative approach to efficiently prioritize highly potential candidates for further experimental consideration. To date, there were six tools developed for predicting PBSs, including Scansite [22,23], SMALI [24], NetPhorest [20], GPS-Polo [25], NetSH2 [26], and 14-3-3-Pred [27] (Table S1). Scansite is the earliest online service that can predict both PK-specific p-sites and PBSs, and its latest 4.0 version contained 16 position-specific scoring matrices (PSSMs) derived from OPLS data for predicting potential PBSs [22,23]. Using the OPLS results, SMALI [24] and NetSH2 [26] constructed 76 PSSMs and 70 artificial neural network (ANN) models to predict PBSs of various SH2 domains, respectively. Previously, we collected 56 known PBSs specifically recognized by PBDs in PLKs and designed a tool named GPS-Polo, using a group-based prediction system (GPS) 2.2 algorithm [25]. In 2008, Miller et al. developed a comprehensive predictor named NetPhorest, which constructed 63 individual PSSMs or ANN models to predict PBS for 104 individual PPBDs belonging to five families [20]. Through collecting 328 14-3-3-binding p-sites, 14-3-3-Pred averaged the scores generated by three algorithms including PSSM, ANN and support vector machine (SVM) [27]. More details on these computational programs, including the data sources, sizes of training data sets, algorithms, web links, and window sizes for encoding PBS peptides were shown, as well as original references (Table S1). 

In this work, we manually collected 4458 experimentally identified PBSs in 950 PPBD-binding proteins (PPBPs) that interact with 268 PPCPs from 12 eukaryotic species (Table S2). We classified these known PBSs into a hierarchical structure with three levels, including group, family, and single PPBD cluster, based on the annotations of PPCPs [28]. With a hypothesis that PPBDs in the same family/cluster might recognize similar sequence motifs in substrates, we considerably improved our previously developed GPS algorithm [25,29,30], and adopted a deep learning plus transfer learning for model training. Then we developed a new online service named GPS-PBS, which implemented 138 predictors for 122 PPBD clusters belonging to twi groups and 16 families. In total, GPS can predict PBSs for 159 human PPCPs. By comparison, our results demonstrated that GPS-PBS showed a highly competitive accuracy against other exiting tools. Using GPS-PBS, we conducted a large-scale prediction to computationally annotate potential PPBDs from a mammalian phosphoproteomic data set and observed that various PPCPs and PKs are involved in synergistically orchestrating a number of important pathways. Taken together, we anticipate that GPS-PBS can be a helpful tool to prioritize highly potential candidates for further experimental consideration. For convenience, the online service of GPS-PBS was implemented in PHP and JavaScript, and freely available for academic research [31].

## 2. Materials and Methods

### 2.1. Data Collection and Preparation

From PubMed, we initially used multiple keyword combinations, such as “((phosphorylation) AND site) AND bind”, “(recognize) AND phosphorylation site” and “((phosphorylation) AND site) AND protein interaction domain”, to search experimentally identified PBSs by carefully checking abstracts or full texts of the scientific literature published before January 1, 2018. Additionally, we further obtained 2888 unambiguous PBSs from PepCyber:P~PEP [17], whereas PMIs not involved in the interactions between PPBDs and p-sites were discarded. We pooled the two data sets together, and mapped PBSs to their primary protein sequences downloaded from the UniProt database [32] to pinpoint the exact binding positions. After redundancy clearance, we in total obtained 4458 PBSs in 950 PPBPs (Table S2).

Here, we defined a PBS peptide PBP(*m*, *n*) as a S/T/Y residue flanked by *m* residues upstream and *n* residues downstream. As previously described [33], we adopted PBP(10, 10) for model training and parameter optimization in a rapid manner. For PBSs located at N- or C-terminals, we added one or multiple special characters “*” to complement the full PBP(10, 10) entries. To obtain a general benchmark data set for the initial deep learning, PBP(10, 10) entries derived from known PBSs were taken as positive data, while PBP(10, 10) peptides around other non-binding S/T/Y residues were regarded as negative data. Two benchmark data sets were separately generated for pS/pT- and pY-interacting PPBDs. For each benchmark data set, we separately cleared the redundancy of positive data and negative data at the peptide level, and reserved only one PBP(10, 10) item if multiple identical entries were found. For further transfer learning, a benchmark data set was generated for each PPBD family or single PPBD based on the classification information, and redundancy clearing was also conducted. 

### 2.2. Performance Evaluation

Four measurements including accuracy (*Ac*), sensitivity (*Sn*), specificity (*Sp*), positive predictive value (*PPV*), negative predictive value (*NPV*), and Mathew Correlation Coefficient (*MCC*) were used to evaluate the prediction performance, and defined as below:

(1) $$Ac=\frac{TP+TN}{TP+FP+TN+FN},Sn=\frac{TP}{TP+FN},\;Sp=\frac{TN}{TN+FP},$$

(2) $$PPV=\frac{TP}{TP+FP},\;NPV=\frac{TN}{TN+FN},$$

(3) $$MCC=\frac{\left(TP\times TN\right)-\left(FN\times FP\right)}{\sqrt{\left(TP+FN\right)\times \left(TN+FP\right)\times \left(TP+FP\right)\times \left(TN+FN\right)}}.$$

The 4-, 6-, 8-, and 10-fold cross-validations were performed for PPBD families or single PPBDs with ≥30 PBSs, respectively, whereas the LOO validation was performed for other PPBD clusters. For each validation, the corresponding *Sn*, *Sp*, *Ac* and *MCC* values were calculated. The ROC curves were plotted based on *Sn vs*. 1-*Sp* values, and the AUC scores were calculated.

### 2.3. An Improved GPS Algorithm

**Figure 1:**
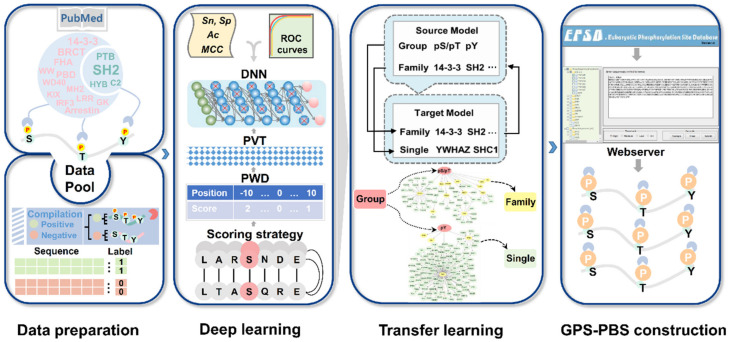
The full experimental procedure of the study. From the scientific literature and the PepCyber:P~PEP database [17], we collected 4458 known PBSs in 950 PPBPs that interact with 268 PPCPs, and compiled a benchmark data set containing PBSs for 2 groups, 16 families, and 122 single PPBD clusters. Then, we updated our previously developed GPS algorithm [25,29,35], and implemented a deep learning plus transfer learning strategy to obtain family- and single PPBD cluster-based models from two general models for the pS/pT and pY groups. Finally, we developed a new online service named GPS-PBS, which constructed 138 models to hierarchically predict PBSs for 158 human PPCPs.

In this study, we considerably improved our previous GPS algorithm that contained three parts, including a basic scoring strategy, a position weight determination (PWD) method, and a peptide-to-vector transformation (PVT) approach ( Figure 1; Figure 3). The algorithm was described as below:

(*i*) The basic scoring strategy. Initially, the average similarity score (*S*) between a PBP(10, 10) item *A* and the whole positive data set was defined as:
  (4) $$S=\frac{1}{N}\overset{K}{\underset{j=1}{\sum }}(\overset{N}{\underset{i=1}{\sum }}M\left[A_{j},\;P_{ij}\right])\times W_{j},$$
  where *K* is the length of the PBP(10, 10) peptide and equal to 21, and *N* is the number of positive PBP(10, 10) entries. *P_ij_* is the amino acid residue at position *j* of a positive PBP(10, 10) *P_i_* (*i* = 1, 2, 3, …, *N*). *W_j_* is the weight value of position *j*, and *M* denotes an amino acid substitution matrix BLOSUM62 used in this study.
(*ii*) PWD. In this part, the weight value of each position in the PBP(10, 10) item was initialized as 1. Then, we adopted the original PLR algorithm with the LASSO regularization to optimize the weight values of different positions. The 10-fold cross-validation was conducted, and the corresponding AUC value was calculated. To further enhance accuracy and avoid overfitting, we added two methods including random mutation and random zeroing. In the step of random mutation, we randomly chose a weight value for +1 or −1 per time, and re-calculated the AUC value. The manipulation was accepted if the AUC value was increased. In the step of random zeroing, a weight value was randomly selected and set to 0, and the manipulation was adopted if the AUC value was increased. The two steps were iteratively repeated, and the optimal *W_j_* vectors were determined if the AUC value was not increased any longer, with a numeric criterion of 1*10^-5^ after 50 iterations. The PLR algorithm was implemented in Python 3.6 with Scikit-learn 0.21 [34].
*iii*) PVT. Given the final *W_j_* vectors, the average similarity score (*S_ab_*) of residue *a* in the given PBP(10, 10) item *A* and the amino acid *b* in the positive data set was defined as below:
  (5) $$S_{ab}=\frac{1}{\sum_{j=1}^{21}D_{j}}\sum_{j=1}^{21}D_{j}\times M\left[a,b\right]\times W_{j},$$
  where *D_j_* is the number of *ab* amino acid pairs at position *j*. For the 21 types of pseudo amino acids listed in an alphabetical order (*A*, *C*, *D*, …, *Y*, *), there were a number of [21*(21+1)]/2 = 231 unique *S_ab_* scores (*S_ab_* = *S_ba_*). These scores reflect the position-weighted similarity of amino acids between the given PBP(10, 10) item and all positive PBP(10, 10) entries. Thus, PVT represents a PBP(10, 10) item into a 231-dimensional vector, as below:
  (6) $$V={\left(S_{AA},S_{AC},S_{AD},\cdots ,\;S_{**}\right)}_{231}.$$

### 2.4. The Deep Learning Framework

For training a general model to predict PBSs recognized by pS/pT- or pY-interacting PPBDs, a framework of seven-layer deep neural networks (DNNs) was implemented, containing one input layer, five fully connected (hidden) layers and one output layer. Each layer consisted of a number of computational units called neurons. To avoid over-fitting, which frequently occurs in deep learning algorithms, the dropout method was used by randomly dropping nodes from the five hidden layers if the *Ac* value was increased. In each layer, both internal feature representations and computational outcomes were connected and propagated by neurons. The input layer receives a data matrix per time, in which each line represents a 231-dimensional vector of a unique PBP(10, 10) item. The five hidden layers were mainly used for feature extraction and representation. A rectified linear unit (ReLU) activation function was adopted to activate the outcome of a neuron, and defined as below:

(7) $$ReLU\left(x\right)=\left\{\begin{array}{c} x,\;\;x\geq 0\\ 0,\;\;x<0 \end{array}\right.,$$

where *x* was the weighted sum of a neuron. The output layer contains two sigmoid neurons to calculate a score for a given PBP(10, 10) item *y,* defined as below:

(8) $$P\left(y\right)=sigmoid\left(y\right)=\frac{1}{1+e^{-y}}.$$

The *P*(*y*) value denotes the probability score of a PBP(10, 10) item to be a real PBS. 

A lab computer with an Intel(R) CoreTM i7-6700K@ 4.00 GHz central processing unit (CPU), 32 GB of RAM, and a NVIDIA GeForce GTX 960 core were used for training the two general models for predicting pS/pT- and pY-interacting PPBDs, respectively. The training process was implemented in the Keras 2.1.5 library with the tensorflow 1.10.0 backend. Training parameters including dropout ratio, degree of momentum, learning rate, mini-batch size, number of nodes, and strength of parameter regularization were simultaneously optimized to reach an optimal AUC value from the 10-fold cross-validations.

### 2.5. A Permutation Test to Detect Significant Associations of PPBDs and PKs

Here, we defined a doubly regulated p-site (DRP) as a p-site that was predicted to be phosphorylated by at least one PK and also interact with at least one PPBD, at the family level. For a PPBD family *K*, the number of its interacting PBSs was counted as *m_K_*. For a PK family *L*, the number of its substrate p-sites was counted as *n_L_*. The number of DRPs for *K* and *L* on the same p-sites was counted as *x*. Then, the GPS-PBS predictions were not changed, whereas the prediction results of GPS 5.0 in 638,909 p-sites were randomly permutated. The number of p-sites modified by *L* remained to be *n_L_*, whereas the DRPs for *K* and *L* was re-counted as *x’*. Such a permutation test was repeated 10,000 times, and the results were modeled in a Gaussian distribution. The *p*-value was calculated based on the proportion of *x’* ≥ *x*.

### 2.6. Implementation of the Web Service

To estimate the FPR values, we randomly retrieved 10,000 PBP(10, 10) items from Swiss-Prot protein sequences downloaded from UniProt to construct a near-negative data set. Then, GPS-PBS was used for a prediction. For each PPBD family or single PPBD, the theoretical FPR value was calculated as the predicted number against the 10,000 PBP(10, 10) items. Such a process was repeated 20 times, and the average value was determined as the final FPR. Again, the FPR values were separately estimated for pS/pT- and pY-interacting PPBDs. The high, medium and low thresholds were adopted with FPRs of 2%, 6%, and 10% for pS/pT-interacting PPBDs and 4%, 9%, and 15% for PPBDs in the pY group, respectively. We also implemented an “All” option to output all predictions for one or multiple selected predictors. GPS-PBS was extensively tested on various web browsers including Internet Explorer, Mozilla Firefox, and Google Chrome to ensure its usability. 

## 3. Results

### 3.1. A Deep Learning Plus Transfer Learning Strategy for Predicting PBSs

The full procedure of this study was shown in Figure 1. Through the literature biocuration and public database integration, we obtained 4458 known PBSs involving 950 PPBPs and 268 PPCPs in eukaryotes (Figure 1, Table S2). Then, all PBSs were classified into two groups including the pS/pT group and the pY group, respectively, based on their interacting PPCPs. According to the classification information of PPCPs [28], the PBSs under the pS/pT group were further classified into 12 families, including 14-3-3, BRCA1 carboxyl-terminal (BRCT), forkhead-associated (FHA), kinase-inducible domain interacting domain (KIX), PBD, WW, Mad homology 2 (MH2), WD40, Interferon-regulatory factor 3 (IRF3), Guanylate kinase (GK), Arrestin and leucine-rich repeat (LRR), whereas the dataset of the pY group was also categorized into four families, including SH2, phosphotyrosine-binding (PTB), protein kinase C conserved region 2 (C2), and Hakai phospho-tyrosine binding domain (HYB) (Figure 1). Besides the group and family levels, we also considered the classification of PBSs at the single PPBD level, and PBSs recognized by orthologous PPBDs conserved in different species were merged into the same cluster of single PPBDs. Only single PPBDs with ≥3 known PBSs were reserved. After redundancy clearance, a benchmark data set was generated with 2 groups, 16 families and 122 single PPBD clusters.

For the prediction of PBSs specifically recognized various PPCPs, here we improved our GPS algorithm [25,29,30] to contain three parts, including a basic scoring strategy, a position weight determination (PWD) method, and a peptide-to-vector transformation (PVT) approach (Figure 1). The scoring strategy measured the sequence similarity of PBSs together with their flanking peptides, whereas PWD optimized the weight values of different positions in peptides. To enable model training with a deep learning framework of seven-layer DNNs, we developed a new method named PVT to transform the single similarity score of a PBS peptide into a 231-dimensional vector. Two DNN models were trained for the pS/pT and pY groups, respectively. To obtain family-based models, transfer learning was adopted by using the two general models derived from DNNs, and the family-specific data was used to fine-tune the model of each PPBD family (Figure 1). Again, using the family-based models, we further used transfer learning to obtain the model for each single PPBD cluster (Figure 1). In order to provide an applicable tool for the research community, we constructed a new online service named GPS-PBS, which can hierarchically predict PBSs for 159 human PPCPs belonging to 2 groups, 16 families, and 122 single PPBD clusters, respectively (Figure 1).

### 3.2. The Data Statistics and Analysis of Known PBSs

**Figure 2:**
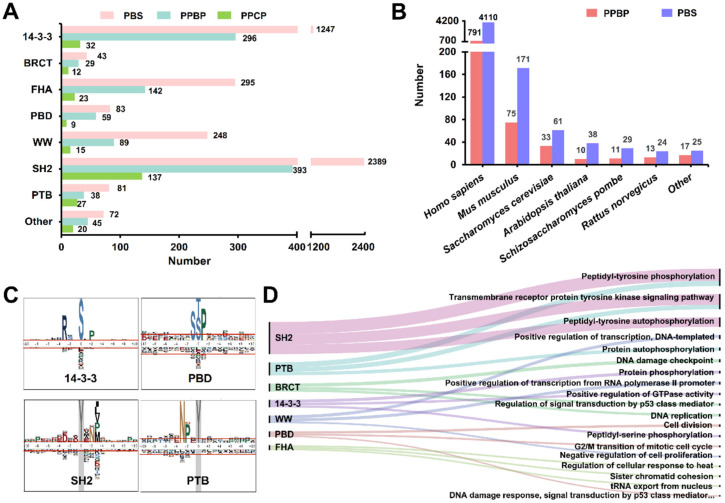
The statistics and analysis of the benchmark data set. (**A**) The numbers of known PBSs, PPBPs and PPCPs for several major PPBP families. (**B**) The distribution of numbers of known PPBPs and PBSs in the 12 eukaryotic organisms. (**C**) The sequence logos of PBS peptides for four PPBD families including 14-3-3, PBD, SH2 and PTB. (**D**) The GO-based enrichment analysis of human PPBPs belonged to seven families by WocEA [30] (*p*-value < 10^−5^).

From the 4458 known PBSs, the numbers of PBSs, PPBPs and PPCPs were counted for several major families such as 14-3-3, BRCT, FHA, PBD, WW, SH2, PTB and other families (Figure 2A). We observed that the SH2 family, the first discovered PPBD family that had been extensively studied [5], had most data with 2389 experimentally validated PBSs involving 137 individual PPCPs and 393 PPBPs. The 14-3-3, FHA and WW families had smaller data sizes, with 1247, 295, and 248 known PBSs (Figure 2A). In our data set, known PPBPs with corresponding PBSs were collected from 12 eukaryotic organisms, including *Homo sapiens*, *Mus musculus*, *Saccharomyces cerevisiae*, *Schizosaccharomyces pombe*, *Rattus norvegicus*, *Arabidopsis thaliana* and other species (Figure 2B). In total, there were 4110 (92.19%) unique PBSs of 791 proteins in *H. sapiens*, and the result indicated that the most of PBS-related experiments were conducted in human cells (Figure 2B).

Furthermore, we adopted pLogo [36], a widely used motif logo generator, to analyze the sequence preferences of PBSs for each PPBD family. The results of several typical families such as 14-3-3, PBD, SH2 and PTB families were present (Figure 2C). For the 14-3-3 family, we found that arginine (R) and proline (P) residues were statistically over-represented at positions -3 and +2. Although with a less significance, R residues were also enriched at positions -2, -4 and -5, while S residues preferred to occur at the position -2. Our result was highly consistent with a previously reported motif RxRSxpSxP for the 14-3-3 family [37]. For the SH2 family, asparagine residues frequently occurred at the position +2, whereas hydrophobic residues such as isoleucine (I), leucine (L), valine (V), P and methionine (M) were enriched at the position +3, and the result was also well consistent with known sequence patterns of PBSs interacting with SH2 proteins [38,39]. To demonstrate whether the sequence diversity of PBSs for different PPBDs was also taken into account in our classification, the sequence logos were visualized for four single PPBD clusters including YWHAZ, YWHAB, SFN, and YWHAE of the 14-3-3 family (Figure S1A), and four single clusters including GRB2, SHC1, SRC, and PIK3R1 of the SH2 family (Figure S1B). From the results, it could be found that although the sequence profiles of four 14-3-3 members were highly similar, the significance of P at the position +2 of YWHAZ was lower than the other three clusters (Figure S1A). Additionally, T residues were only enriched in PBSs of YWHAZ and SFN at the position -2 (Figure S1A). For the members of the SH2 family, the sequence diversity of PBSs is much higher (Figure S1B). PBSs of GRB2 follow a sequence motif of YXN, whereas the sequence pattern of PIK3R1 is YXXM [40]. The sequence profiles of PBSs for GRB2 and PIK3R1 are highly different with SHC1 and SRC (Figure S1B). Taken together, our results suggested that both the sequence similarity and diversity of PBSs in the hierarchical classification.

Using the 776 known human PPBPs belonging to seven families (Table S2), we conducted an enrichment analysis based on Gene Ontology (GO) annotations with the hypergeometric test [30,41] (Figure 2D, *p*-value < 10^−5^). The top three mostly enriched GO biological processes were selected and visualized for each family. For the pY group, we observed that PPBPs of the SH2 and PTB families were significantly enriched in tyrosine phosphorylation-associated processes, such as peptidyl-tyrosine phosphorylation (GO:0018108), transmembrane receptor protein tyrosine kinase signaling pathway (GO:0007169) and peptidyl-tyrosine autophosphorylation (GO:0038083). The results were highly consistent with experimental studies, which demonstrated that PPBPs played a critical role in reading the dynamic signals of pY phosphorylation networks [4].

For the pS/pT group, the enrichment GO biological processes such as protein phosphorylation (GO:0006468), positive regulation of GTPase activity (GO:0043547) and peptidyl-serine phosphorylation (GO:0018105) for the 14-3-3 family demonstrated that their PPBPs were highly involved in regulating pS/pT phosphorylation events. Further analyses of BRCT, FHA, PBD and WW families in the pS/pT group indicated that their PPBPs were highly involved in regulating various types of DNA-associated processes such as positive regulation of transcription, DNA-templated (GO:0045893), DNA damage checkpoint (GO:0000077) and sister chromatid cohesion (GO: 0007062) (Figure 2D). These results were not only consistent with previous reports [1,42], but also provided valuable information for further deciphering regulatory roles of human PPBPs.

### 3.3. Development of GPS-PBS to Predict PBSs Recognized by Various PPBDs

Previously, we developed the GPS 2.0 algorithm for the prediction of kinase-specific p-sites [29]. Later, we updated the algorithm into the 2.2 version, and used it to developed a tool named GPS-Polo 1.0 to predict potential PBSs interacting with PBDs in PLKs [25]. Recently, we improved the algorithm and designed a tool named GPS 5.0, which implemented 617 individual predictors for computationally detecting potential p-sites of 479 human PKs [30]. Based on a hypothesis of similar peptides potentially exhibiting similar functions, the basic scoring strategy for measuring the peptide similarity was reserved in all versions of the GPS algorithm. For performance improvement, GPS 5.0 adopted two additional methods including PWD and scoring matrix optimization (SMO), and the latter was developed for obtaining an optimal matrix from an initial amino acid substitution matrix, e.g., BLOSUSUM62 [30].

**Figure 3:**
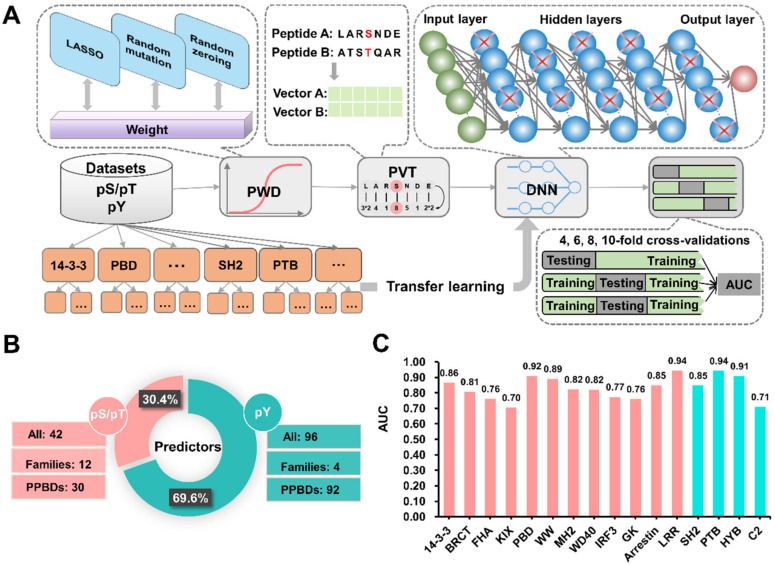
The implementation and accuracy of GPS-PBS. (**A**) The procedure of model training in the updated GPS algorithm, in which the basic scoring strategy, PWD and PVT, were implemented to transform single similarity scores into 231-dimensional vectors. A deep learning framework of seven-layer DNNs was adopted to train two models for the pS/pT and pY groups, respectively. Then, transfer learning was adopted to obtain models in family and single PPBD cluster levels. (**B**) The statistics of individual predictors in the pS/pT and pY groups. (**C**) For the 16 PPBD families, the AUC values were calculated from the 10-fold cross-validation or the LOO validation.

In this work, we hypothesized that the amino acid residues in each position around PBSs might be generally and differentially important for the recognition of PPBDs. For developing GPS-PBS, both the scoring strategy and PWD were reserved, and we also added a new method named PVT (Figure 3A). In the step of PWD, the weight values of different positions for PBSs and their corresponding flanking peptides were computationally optimized by a refined penalized logistic regression (PLR) algorithm, with the least absolute shrinkage and selection operator (LASSO, L1 regularization) and two additional approaches including random mutation and random zeroing (Figure 3A). Using BLOSUM62, PVT automatically assigned a unique 231-dimensional vector for each PBSs (Figure 3A). Then, the deep learning plus transfer learning strategy [43] was adopted for model training (Figure 3A). In total, we obtained 138 individual models for 16 PPBD families and 122 single PPBD clusters (Table S3).

In GPS-PBS, we constructed 42 predictors including 12 family-based and 30 single PPBD cluster-based predictors for the pS/pT group (Figure 3B). For the pY group, we implemented 96 individual predictors including four family-based and 92 single PPBD cluster-based predictors, which accounted for 69.6% of the total predictors (Figure 3B). It should be noted that GPS-PBS could predict PBSs for up to 10 families of PPBDs for the first time, including FHA, KIX, MH2, WD40, IRF3, GK, Arrestin, LRR, HYB, and C2. To evaluate the accuracy and robustness of each model, 4-, 6-, 8-, and 10-fold cross-validations were performed for 36 PPBD families or single PPBD clusters, whereas the leave-one-out (LOO) validations were conducted for remaining models (Tables S4 and S5). The receiver operating characteristic (ROC) curves illustrated, and the area under ROC (AUC) values were computed. From the 10-fold cross-validation or LOO validation, we found that AUC values ranged from 0.70 to 0.94 for the 16 PPBD families, while the top five families with the highest AUC values were PTB (0.94), LRR (0.94), PBD (0.92), HYB (0.91), and WW (0.89), respectively (Figure 3C). More details on the performance evaluation could be available in Tables S4 and S5. Additionally, the numbers of positive and negative PBP(*m*, *n*) items, as well as *m* and *n* values, were present for each PPDB family or single PPBD cluster. The ratio of negative: positive PBP(*m*, *n*) items ranged from 4.3 (LCP2_SH2) to 204.3 (MDC1_BRCT), indicating the imbalance of positive and negative data in the benchmark data set (Tables S4 and S5).

### 3.4. Comparison of GPS-PBS to Other Existing Tools

**Figure 4:**
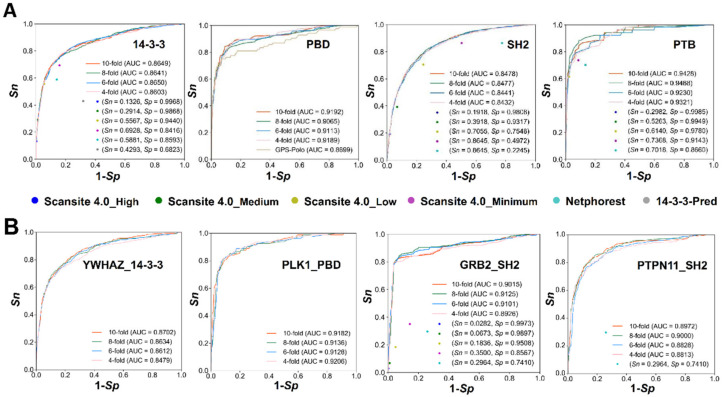
Comparison of GPS-PBS with other existing tools, including Scansite 4.0 [23], NetPhorest [20], 14-3-3-Pred [27], and GPS-Polo 1.0 [25] at (**A**) the family level, and (**B**) the single PPBD cluster level. The ROC curves were illustrated for GPS-PBS, based on 4-, 6-, 8-, and 10-fold cross-validations.

To further demonstrate the superiority of GPS-PBS, we compared it to other existing tool such as Scansite 4.0 [23], NetPhorest [20], and 14-3-3-Pred [27], as well as our previously developed GPS-Polo 1.0 [25]. For simplicity, the results of four PPBD families including 14-3-3, PBD, SH2, PTB were shown (Figure 4A). For each family, we directly submitted the corresponding benchmark data set into these tools to calculate the accuracy values, which were compared with the 4-, 6-, 8-, and 10-fold cross-validations of GPS-PBS. The ROC curves of GPS-Polo 1.0 [25] were presented, whereas the *Sn* and *Sp* values of Scansite 4.0 [23], NetPhorest [20], and 14-3-3-Pred [27] were computed at different or default thresholds provided in these tools. From the results, we found that the accuracy of GPS-PBS in the PPBD family level was higher or at least comparative with these existing tools (Figure 4A).

Moreover, we chose four single PPBD cluster-based predictors including YWHAZ, PLK1, GRB2 and PTPN11 (Figure 4B). It was found that only Scansite 4.0 [23] and NetPhorest [20] also contained single PPBD cluster-based predictors, whereas GPS-PBS achieved a highly competitive accuracy against the two predictors (Figure 4B). In particular, the highly similar results of the 4-, 6-, 8-, and 10-fold cross-validations indicated the robustness of computational models in GPS-PBS.

### 3.5. A Large-Scale Prediction of Potential PBSs from the Phosphoproteomic Data

In a recent study, we developed a comprehensive resource named eukaryotic phosphorylation site database (EPSD), which contained 1,616,804 experimentally identified p-sites in 209,326 proteins collected from 68 eukaryotic species [44]. From EPSD, we obtained 765,779 p-sites including 468,630 pS, 204,997 pT, and 92,152 pY sites of three major mammalians including *H. sapiens*, *M. musculus,* and *R. norvegicus*. These p-sites were detected from low-throughput or high-throughput experiments, whereas the PPBD information for most of the p-sites still remained to be annotated. 

**Figure 5:**
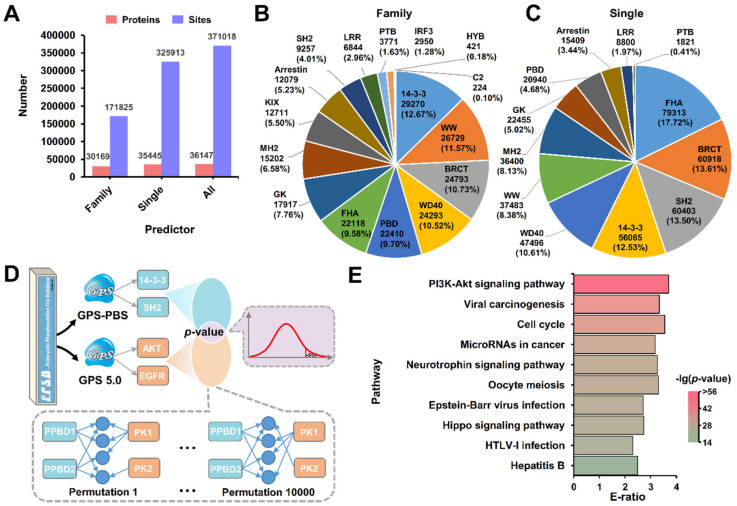
A large-scale prediction of potential PPBDs from a mammalian phosphoproteomic data set. (**A**) From 765,779 mammalian p-sites obtained from EPSD [44], GPS-PBS predicted potential PBSs using family-based and/or single PPBD cluster-based predictors. The family distribution of numbers of potential PBSs were shown for (**B**) the family-based predictions, and (**C**) the single PPBD cluster-based predictions. (**D**) After identification of potential DRPs, the significant associations between PPBDs and PKs in regulating the same p-sites were detected through a permutation test (*p*-value < 10^-10^). (**E**) The KEGG-based enrichment analysis of DRP-containing proteins against all predicted PPBPs.

Using GPS-PBS, we here performed a large-scale prediction of PBSs to annotate potential PPBDs for the mammalian p-sites. The high threshold of GPS-PBS was chosen with false positive rate (FPR) values of 2% and 4% for the PPBDs in the pS/pT and pY groups, respectively. From the results, we found that only 171,825 (22.44%) potential PBSs were predicted at the family level, whereas up to 325,913 (42.56%) p-sites were computationally annotated to potentially interact with at least one PPBD using the single PPBD cluster-based predictors (Figure 5A). In total, there were 371,018 p-sites predicted to be potential PBSs, with a coverage of 48.45% for the mammalian phosphoproteomic data set (Figure 5A). The family distribution of numbers of potential PBSs was analyzed for the family-based predictions (Figure 5B). It could be found that the top three families with most predicted PBSs were 14-3-3 (29,270, 12.67%), WW (26,729, 11.57%) and BRCT (24,793, 10.73%) (Figure 5B). Interestingly, although the SH2 family had the greatest number of known PBSs, we only predicted 9257 PBSs (4.01%) potentially interacting with SH2 proteins, using the family-based predictor. In addition, the distribution of numbers of predicted PBSs in different families was also analyzed for single PPBD cluster-based predictions (Figure 5C). Indeed, single PPBD cluster-based predictions enhanced the PBS annotations for several families such as FHA and SH2 with less numbers of potential PBSs using family-based predictors. Thus, our results indicated that both types of predictions will be helpful for further experimental consideration. 

In dynamic phosphorylation networks, both PKs and PPCPs are important regulators, and the identification of significant associations between the two types of regulators will be helpful for better understanding the mechanisms of phosphorylation. Using GPS 5.0 with the high threshold [30], we predicted 638,909 (83.43%) p-sites to be potentially regulated by at least one PK family. The prediction results of GPS-PBS and GPS 5.0 were compared, and we obtained 158,525 potential DRPs that might be modified by at least one PK and also interact with at least one PPBD, at the family level. We used the identified DRPs to conduct a permutation test (*p*-value < 10^−10^) and identified 124 pairs of significant associations between PPBD and PK families in regulating the same p-sites (Figure 5D, Table S6). In our results, a number of associations between PPBD and PK families have been well documented, such as 14-3-3 and Akt [45], and 14-3-3 and CAMK [45,46]. Thus, our analysis was highly consistent with experimental observations. Using the hypergeometric test (*p*-value < 0.01), an enrichment analysis was performed based on Kyoto Encyclopedia of Genes and Genomes (KEGG) annotations [47] for 1060 DRP-containing proteins regulated by the 124 pairs of PPBD and PK families that shared at least one common KEGG term against the 5269 annotated PPBPs. The top five mostly enriched pathways were PI3K-Akt signaling pathway (hsa04151), viral carcinogenesis (hsa05203), cell cycle (hsa04110), MicroRNAs in cancer (hsa05206) and neurotrophin signaling pathway (hsa04722), indicating that PKs and PPBDs are synergistically involved in regulating these pathways (Figure 5E).

## 4. Discussion

Catalyzed by writers named PKs, p-sites in phosphoproteins frequently exert their functional effects through following recognition by PPBDs to interact with PPCPs, which act as readers and disseminate phosphorylation signals into downstream pathways for regulating cellular activities [5,6,7]. Although over 1.6 million of eukaryotic p-sites have been identified mainly from mass spectrometry-based phosphoproteomic studies, most of their interacting PPBDs remain to be dissected. In contrast with labor-intensive and time-consuming experiments, computational prediction of potential PBSs can greatly narrow down potential candidates and provide useful information for further experimental consideration. To date, >30 tools have been developed to predict regulatory PKs for p-sites [48], whereas only six predictors are available for identifying potential PBSs (Table S1).

In this work, we compiled a high-quality benchmark data set containing 4458 PBSs (Table S2), considerably improved our previously developed GPS algorithm, adopted a deep learning plus transfer learning strategy, and developed a new online service named GPS-PBS for the hierarchical prediction of PBSs specifically recognized by 122 individual PPBD clusters belonging to 2 groups and 16 families (Figure 3C). By comparison, GPS-PBS showed a highly competitive accuracy against other existing tools (Figure 4). It should be noted that there were 4145 known PBSs (93.0%) collected from three mammalians including *H. sapiens*, *M. musculus,* and *R. norvegicus*. Only 313 (7.0%) known PBSs were curated from other species. Thus, although GPS-PBS was designed for a more general purpose, it could be expected that the prediction of PBSs in other species would achieve a lower accuracy, beyond the three mammalians. Additionally, only the sequence similarity of members in different PPBD families or single PPBD clusters were taken into account for model building, and the sequence diversity of individually PPBDs were not directly considered. Thus, the prediction accuracy might be lower for less studied PPBDs or PPBDs without known PBSs.

**Figure 6:**
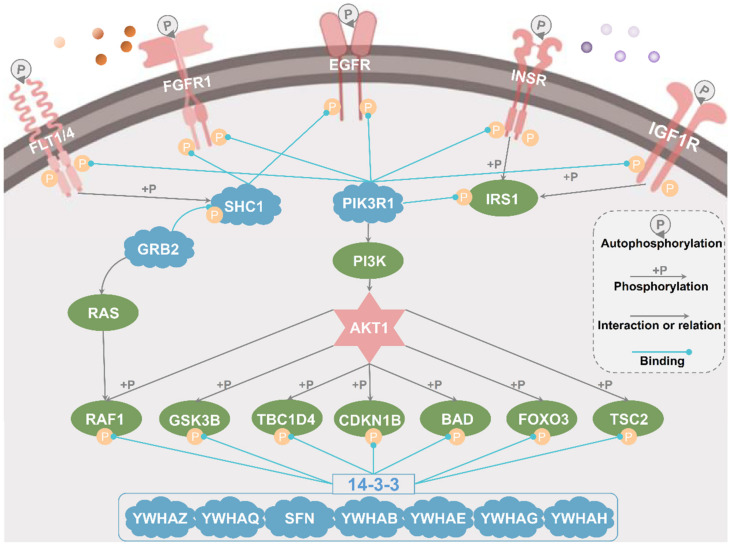
In PI3K-Akt signaling pathway (KEGG ID: hsa04151), various PKs and PPCPs are involved in translating the extracellular stimuli into phosphorylation signals and propagating these signals into downstream pathways.

Using GPS-PBS, we conducted a large-scale annotation of potential PPBDs for 765,779 known mammalian p-sites obtained from EPSD [44], and identified 171,825 (22.44%) potential PBSs potentially interacting with one PPBD at the family level (Figure 5A). Additionally, we used GPS 5.0 [30] and predicted 638,909 (83.43%) p-sites to be phosphorylated by at least one PK family, and further identified 158,525 potential DRPs that might be regulated by both PKs and PPBDs. Through a permutation test, we in total identified 124 pairs of significant associations between PPBD and PK families (Table S6), which synergistically orchestrate a number of important pathways (Figure 5E). For the PI3K-Akt signaling pathway (KEGG ID: hsa04151), the phosphorylation regulations of substrates by PKs and PMIs between PPBPs and PPCPs were illustrated to elucidate how phosphorylation is involved in regulating the pathway (Figure 6). Upon extracellular stimuli, receptor tyrosine kinases (RTKs) such as EGFR, FGFR1, INSR, IGF1R and FLT1/4 can be activated by autophosphorylation, which recruits PTB-containing proteins and SH2-containing proteins such as SHC1 a PIK3R1 [49,50]. The PMIs facilitate the activation of PI3K-Akt signaling pathway. In addition, activated RTKs can also phosphorylate IRS1 and SHC1, whereas the resulting pY residues interact with SH2-containing proteins such as PIK3R1 [51,52] and GRB2 [53] to stimulate the PI3K-Akt signaling pathway. Activated AKT1 can directly phosphorylate a number of proteins such as RAF1, GSK3B, TBC1D4 CDKN1B, BAD, FOXO3 and TSC2, of which PBSs in seven proteins can interact with 14-3-3 proteins to participate in regulating diverse downstream signaling pathways [2,53,54,55,56,57,58] (Figure 6, Table S7). From proteins in the PI3K-Akt signaling pathway, we in total predicted 625 DRPs, of which 28 DRPs have been experimentally validated in previous studies (Table S7). The results not only supported a high accuracy of the DRP inference, but also provided useful information for further experimental design.

In the future, we will continuously maintain GPS-PBS by curating more experimentally identified PBSs if new data becomes available. In GPS-PBS, we adopted PBP(10, 10) for model training, and different combinations of *m* and *n* values of PBP(*m*, *n*) items will be tested later. Moreover, the BLOSUM62 matrix was adopted to measure the peptide similarity of PBP(*m*, *n*) items, and we will test other types of amino acid substitution matrices for performance improvement. The computational models in GPS-PBS will be updated if the GPS algorithm was updated. Besides GPS, other algorithms will be tested and integrated into GPS-PBS if the accuracy can be improved. We anticipate that GPS-PBS can be a useful tool for further exploration of dynamic phosphorylation networks.

## Supplementary Materials

The following are available online at https://www.mdpi.com/2073-4409/9/5/1266/s1, Figure S1: The sequence logos for (A) four single PPBD clusters of the 14-3-3 family, and (B) four single PPBD clusters of the SH2 family, Table S1: A summary of the 6 existing tools for the prediction of PBSs, Table S2: Through the literature biocuration and public database integration, we collected 4458 experimentally PBSs involving 950 PPBPs and 268 PPCPs in 12 eukaryotes, Table S3: GPS-PBS contained 138 predictors for 16 families and 122 single PPBD clusters, Table S4: The performance values of 4, 6, 8 and 10-fold cross-validations for 36 predictors with ≥ 30 positive PBSs, under the high, medium and low thresholds, Table S5: The performance values of the LOO validations for 102 predictors with < 30 positive PBSs, Table S6: The 124 significant associations between PPBDs and PKs through the permutation test (*p*-value < 10^-10^), Table S7: Known DRPs in the PI3K-Akt signaling pathway.
