# Supplementary figures and images for: GPS-PBS: A Deep Learning Framework to Predict Phosphorylation Sites that Specifically Interact with Phosphoprotein-Binding Domains
Source: Cells. 2020 May 20;9(5):1266. doi: 10.3390/cells9051266 (PMC7290655; doi:10.3390/cells9051266)

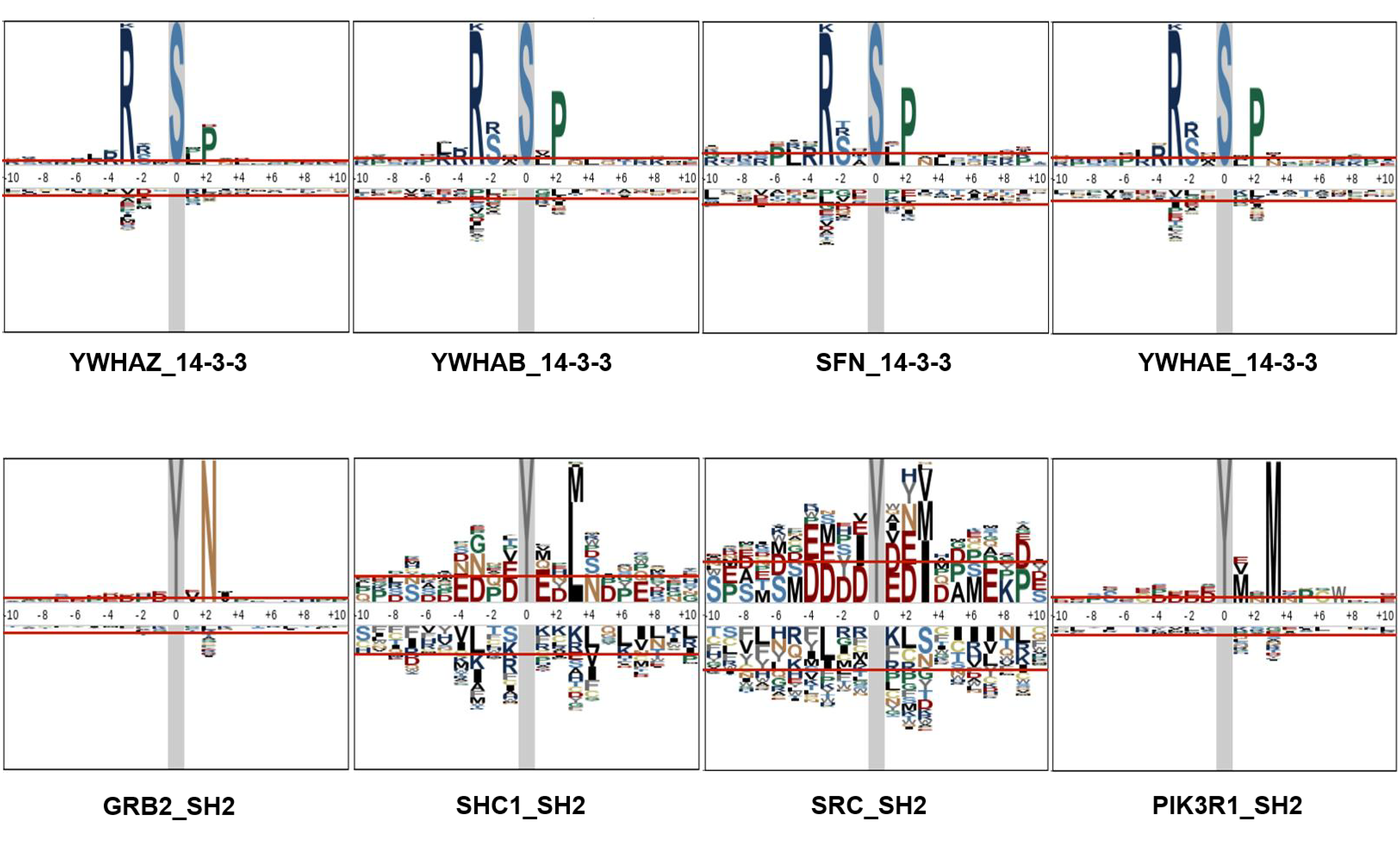

Supplement: Supplementary file 1 [file cells-09-01266-s001.zip › Figure S1.tif]
